# Associations Between Religiosity, Spirituality and Depressive Symptoms Among People Experiencing Homelessness in São Paulo, Brazil: An Observational Study

**DOI:** 10.1007/s11126-025-10163-5

**Published:** 2025-06-09

**Authors:** Felipe Alckmin-Carvalho, Pedro Henrique França Camargo, João Vitor Guedes Neto de Moraes, Patricia Gabriela da Silva, Henrique Pereira, Luciano Magalhães Vitorino

**Affiliations:** 1 Department of Psychology and Education, Faculty of Social and Human Sciences, University of Beira Interior, do Urbanização da Quinta do Pinheiro 4, Covilhã, 6200-552 Portugal; 2 Faculty of Medicine of Itajubá, Itajubá, Minas Gerais Brazil; 3 CIDESD - Research Center in Sports Sciences, Health Sciences and Human Development, Covilhã, Portugal

**Keywords:** Homeless people, Social vulnerability, Depression, Spirituality, Religion and medicine, Coping

## Abstract

Despite the evident relationship between religiosity and spirituality (RS) and symptoms of depression found in different population groups, there is a lack of studies in more vulnerable groups, especially among people experiencing homelessness (PEH) in least developed countries. Our aim was to assess the association between RS and depressive symptoms in PEH in a large Brazilian urban center. This is an observational study in which a sample of PEH participated. We assessed the following variables: religiosity, spirituality, religious-spiritual coping and symptoms of depression. Results: A total of 456 PEH had participated, with an average age of 44.5 (SD = 12.6) years. Of these, 49.6% reported depressive symptoms at clinical level. The linear regression indicated that various aspects related to RS, such as going to a religious temple, having a private spirituality practice, and having a positive religious-spiritual coping related to the positive reinterpretation of stressful situations, were associated with a reduction in the chance of the participants reporting depressive symptoms. However, the presence of negative religious-spiritual coping, related to guilt, feelings of punishment, insecurity, and negative reinterpretation of stressors, increased the risk of symptoms of depression. Our results highlight the relevance of developing and testing the effects of psychosocial interventions that specifically incorporate aspects of RS which produce beneficial impact in the care of PEH who have a religion. This could be a helpful mechanism for reducing symptoms of depression and contributing to the social reintegration of these people. Furthermore, future studies should investigate how to mitigate the potential negative RS beliefs that may contribute to worsening symptoms of depression, to prevent them and mitigate their effects.

**Supplementary Information:**

The online version contains supplementary material available at 10.1007/s11126-025-10163-5.

## Introduction

Depression is a complex mental disorder, resulting from interactions between genetic, environmental and epigenetic factors, and its main symptoms include persistent sadness, loss of interest, fatigue, hopelessness, major changes in appetite and in sleep patterns [5]. In addition, research indicates neuropsychological impairments in executive functions, such as concentration, memory and planning, affecting self-care and work capacity, making depression highly disabling individually and collectively [6–8].

According to the World Health Organization [9], Brazil has the highest prevalence of depression in Latin America. A national study by the Ministry of Health [10] revealed a prevalence of 12.3%, with a higher incidence among women (16.8%) compared to men (7.1%). In the city of São Paulo, South America’s most populous urban conglomerate, a survey found a 16.9% prevalence of depression [11], one of the highest in the world. In the city of Juiz de Fora, for example, the prevalence of mood disorders, especially depression, was 32% [12].

Among the social determinants related to the high prevalence rates of depression among Brazilians living in large urban centers, many are associated with the country’s immense social inequalities and social injustice in the nation, since Brazil is one of the countries with the greatest social inequality assessed by the GINI Index in the world [57]. The main adverse factors that explain, at least in part, the high prevalence of depression among Brazilians living in large cities include high levels of violence, poor housing conditions, unemployment, insufficient income, inefficient public transport, lack of leisure opportunities, lack of green spaces and community centers, poor housing conditions, unemployment, insufficient income and inefficient public transport.

In addition, in large urban centers such as São Paulo, the phenomenon of gentrification has substantially increased the time spent by people of low socioeconomic status in commuting to work, since affordable housing are increasingly distant from the centers of metropolises, where most job opportunities are concentrated [58]. The lack of time, in turn, reduces the likelihood of individuals engaging in self-care behaviors and in leisure opportunities, crease the risk of sedentary lifestyles, obesity, metabolic syndrome, sleep deprivation and consumption of fast food and ultra-processed foods, factors that are known to increase the risk of depression [59-63]. Finally, several studies indicate that stress and anxiety associated with all variables described above, when experienced chronically, alter the individual’s neurophysiology and substantially increase the risk of depression [64, 65].

Socially marginalized populations, such as people experiencing homelessness (PEH), living in large cities in Brazil, face additional adverse conditions such as stigma, lack of belonging, loneliness, lack of social support, food insecurity, exposure to violence, and to adverse weather conditions and lack of access to health and education services. All these additional adverse factors related to homelessness increase the vulnerability of PEH to psychiatric disorders such as anxiety disorders, psychotic disorders, affective disorders, substance abuse disorders, post-traumatic stress disorder and, most commonly, depression [2–4], underlining the social determinants of mental health problems in this population. In fact, a recent review found that PEH were 4 to 5 times more likely to have symptoms of depression compared to those with adequate housing [13]. Depression, especially among homeless people, increases the risk of substance abuse as a coping mechanism for psychological distress, hindering social reintegration and aggravating depression in the long term [18, 19].

Studies carried out in general population indicate that Religiosity and Spirituality (RS) emerge as potential factors to moderate the magnitude and impacts of depression, offering support in coping with adversity [20, 21]. Studies investigating the relationship between RS and mental health have shown encouraging results [22, 23], and psychosocial interventions adapted to faith, such as Cognitive-Behavioral Therapy (CBT), have been shown to be effective in reducing the severity of symptoms of mental disorders in the general population [24, 25]. A systematic review identified a significant association between RS and the reduction of depressive symptoms, with 49% of participants reporting improvement [26].

Despite this, there is a scarcity of studies on the relationship between RS and depressive symptoms in PEH in Latin America, and particularly in Brazil. The country has a cultural and religious predominance of Catholics and Protestants [27] and a continuous increase in the vulnerable population experiencing homelessness [28]. The Global Religion Report [29] revealed that Brazil leads in prevalence of religious belief, with 89% of participants believing in a higher being and 76% having a religion [29].

Given this scenario, we investigated the relevance of RS to the mental health of PEH in Brazil. Our hypothesis is that RS is associated with a reduction in depressive symptoms in this population. We believe that sociodemographic and clinical variables, as well as religious and spiritual coping strategies, influence this process. Our aim is to assess the association between RS and depressive symptoms in PEH a large Brazilian city. We believe that the results will contribute to scientific knowledge on the intersection between mental health, religiosity and spirituality in contexts of social vulnerability. Addressing this issue in a diverse cultural and social context could provide subsidies for clinical, social and public policy interventions that are more effective and sensitive to the needs of this population. We also hope to sensitize professionals to the importance of the spiritual dimension in the assessment and treatment of homeless people, promoting a more holistic and person-centered approach.

## Methods

### Study Design

This is an observational and cross-sectional study in which PEH answered questionnaires, read by trained interviewers, on religiosity, spirituality, and sociodemographic and clinical variables. This study is part of a multidimensional research project on PEH in São Paulo, southeastern Brazil. It addresses associations between spirituality and religiosity and several mental health outcomes, including suicidal ideation, alcohol and substance use and depressive symptoms.

### Location

The research took place in São Paulo. We chose this city because it is the most populous in the country, with around 11.45 million inhabitants [30]. The São Paulo Metropolitan area, made up of 39 municipalities, has approximately 20 million inhabitants [30]. We also chose to focus on São Paulo because it is the Brazilian capital city with the highest number of people experiencing homelessness in the country. In 2023, São Paulo had 54,812 PEH [31]. Finally, the choice of São Paulo was related to the concentration of PEH in specific areas, such as the city center, which facilitated the logistics of data collection. The research was carried out in the Praça da Sé, a location with a large circulation of people, known for its high crime rate, located in São Paulo downtown.

### Data Collection

Data was collected in 2019, in public places such as public squares and streets in Praça da Sé. Due to the vulnerability of the target population, the data was collected during the activities of the NGO “Doutores do Mundo” (Doctors of the World) [33], which assists PEH. The data collection team included three fifth-year medical students from the Faculty of Medicine of Itajubá, and a nurse with a PhD in Public Health and extensive experience in Primary Health Care. The students received training from the lead researcher, including theoretical lessons and a simulated practical exercise with a pilot application of the questionnaires. The NGO’s coordinator, a neurologist and researcher, supervised the entire process. PEH responded to the items on the questionnaires, which were read by trained interviewers without further interpretation, allowing participants to choose the options that best reflected their experiences by responding verbally. This strategy aimed to make the process more accessible, since a significant percentage of PEH in Brazil may have difficulties with reading. The average participation time for each PEH was 30 minutes. If there were any doubts about the inclusion of the participants, the NGO coordinator decided whether to include or exclude them from the survey, based on previous inclusion and exclusion criteria described.

### Participants

Participants were people aged 18 or over, who had been homeless for at least six months (a period of time that we consider sufficient for PEH to come into contact and feel the mental health damage related to the condition of extreme social vulnerability), who were able to understand and respond verbally in Portuguese and who voluntarily consented to take part in the study. People with serious physical or mental health problems (e.g., psychosis, confusion, disorientation), those under the influence of psychotropic substances, individuals with fragile health conditions, and cognitive impairments, or any other neurologic condition that could impair their ability to understand or accurately answer the questions were excluded to ensure the reliability of the responses. All exclusion criteria were evaluated by at least one of the neurologists associated with the research project, through clinical evaluation.

### Variables and Instruments

#### Dependent Variable

Depressive symptoms were assessed using the Patient Health Questionnaire 9 (PHQ-9) [69], validated in Brazilian Portuguese [32]. The PHQ-9, made up of 9 items, assesses the frequency of depressive symptoms in the last two weeks, with scores ranging from 0 (not at all) to 3 (almost every day). Total scores can range from 0 to 27 points. Higher scores indicate more symptoms of depression. In this study, a cutoff point higher than 10 points was adopted to indicate the presence of significant depressive symptoms (moderate, moderately severe, or severe). This cut-off point is associated with high sensitivity (88%) and specificity (88%) compared to the clinical diagnosis of Major Depression [34]. PHQ-9 showed excellent international reliability in a previous Brazilian study (α ⩾ 0.80) [32] and in the present study, internal consistency was very good (α = 0.87).

#### Independent Variables

General data on religiosity and spirituality: general questions were asked about the participant’s beliefs as follows: belief in God and superior being (yes or no), do you have any specific religion (yes or no), do you go to a religious temple? (yes or no), levels of religiosity/spirituality (very religious and very spiritual, very religious and not very spiritual, not very religious and very spiritual, not very religious and not very spiritual); Importance of religion to your life being 0 = not at all important, 1 = a little, 2 = somewhat, 3 = quite, 4 very and; Importance of spirituality to your life being 0 = not at all important, 1 = a little important, 2 = somewhat important, 3 = quite important, 4 very important).

Sociodemographic information: gender (male or female), education (never studied or up to 8 years of studied); marital status (single, married, separated, or widowed), having children (yes or no), state of origin (São Paulo city, São Paulo countryside, outside the state of São Paulo, or outside Brazil), chronic diseases diagnosed in lifetime (yes or no), diagnosed with sexually transmitted infection in life (yes or no) and education (never studied, up to 8 years of study, > 8 years of study). Alcohol and illicit drugs consumption was assessed through the following dichotomous questions: “*Over the past 30 days*, *have you consumed alcoholic beverages at least once a week?” (yes or no); and “Over the past 30 days*, *have you used any illicit drugs (such as marijuana*, *cocaine*, *crack) at least once a week” (yes or no)*.

Religiosity: was assessed through the Brazilian version of The Duke Religious Index (DUREL), called P-DUREL [67]. The scale is based on five items, subdivided into three groups: (I) Organizational Religiosity—OR (frequency to religious gatherings, a 6-point Likert scale, with 1 = never and 6 ⩾ once a week), (II) Non-Organizational Religiosity—NOR (frequency to private religious activities, ranging from 1 = rarely or never to 6 ⩾ once a day), and III) Intrinsic Religiosity—IR (full and individual experience of religiosity, a 5-point Likert scale, ranging from 1 = definitely not true to 5 = totally true). The higher the scores, the higher the levels of religiosity. The Brazilian version has a good internal consistency: α = 0.732 [67]. In the present study, the internal consistency was very good (α = 0.845).

Spiritual Religious Coping (SRC): was assessed through the Spiritual Religious Coping (SRC) Scale (Brief-SRC 14 items), Brazilian version [68]. This scale is divided into positive SRC, with seven items (e.g. ‘I tried to put my plans into action with God’s help’ and ‘I tried to see how God could strengthen me in this situation’), and negative SRC, with seven items (e.g. ‘I kept wondering what I did for God to punish me’ and ‘I came to the conclusion that evil forces acted for this to happen’). The SRC evaluates the way respondents experience and behave when faced with religiosity, which can be either a positive or a negative re-signification of the stressor. Responses were provided on a 5-point Likert scale, ranging from 1 (never) to 5 (very much). Total scores can range between 14 and 70 points. Positive and negative SRC can range between 7 and 35 points, each. The higher the scores, the higher the levels of positive SRC and negative SRC. In the validation study, the positive SRC and negative SRC showed Cronbach’s alpha of 0.884 and 0.845, respectively [68]. In this study, Cronbach’s alpha of the positive SRC dimension was 0.938 and negative SRC was 0.871.

Spirituality: was assessed through the *Functional Assessment of Chronic Illness* Therapy-Spiritual *Well-Being Scale* (FACIT-Sp-12), in the version validated for the Brazilian population [66]. It is a self-assessment scale consisting of 12 criteria, subdivided into three dimensions: Meaning, Peace, and Faith. Each domain has a possible score range of 0 to 16, with total scores ranging from 0 to 48. Higher scores indicate a higher level of spirituality. Participants rate the items using a 5-point Likert scale, ranging from 0 (‘not at all’) to 4 (‘enormously’). Respondents were asked to reveal how true an item had been for them in the past week. Some examples of statements include ‘I feel at peace’ (Peace), ‘I have a reason to live’ (Meaning), and ‘I find comfort in my faith or spiritual beliefs’ (Faith). In the Brazilian validation, the FACIT-Sp 12 showed good reliability within the Brazilian context (α =.890) [66], as well as in this study (α =.770).

### Statistical Analysis

Data were managed using the Statistical Package for the Social Sciences - SPSS^®^ version 27. Descriptive analysis of sociodemographic data was performed, including calculation of mean and standard deviation for age and presentation of absolute values ​​and proportions of all other sociodemographic variables. To evaluate the outcomes of depressive symptoms, religiosity, spirituality and religious and spiritual coping, mean, standard error and 95% confidence interval calculations were performed. We ran unadjusted and adjusted linear regression models for sociodemographic and health variables with *p* < 0.10. In the adjusted model, depressive symptoms were controlled for variables with *p* < 0.10, including age (*p* = 0.079), gender (*p* < 0.001), education (*p* < 0.001), state of origin (*p* = 0.012), chronic diseases (*p* < 0.001), sexually transmitted infections (*p* < 0.001), alcohol use (*p* = 0.001), and drug use (*p* < 0.001). We adopted *p* < 0.05 as significant, with a 95% CI. Statistical power was calculated based on the hypothesis that depressive symptoms among homeless individuals might vary depending on religiosity or spirituality. Using G*Power 3.1.9.7 software, with an effect size of d=-0.1814, α = 0.05, *n* = 456, and seven predictors, the statistical power was determined to be 99%.

### Compliance with Ethical Standards

The project was approved by the Research Ethics Committee of the Faculty of Medicine of Itajubá, Brazil (registration #3.152.988). All participants received detailed information about the study and signed the Informed Consent Form. Our study was conducted in accordance with the Declaration of Helsinki. Authors have no conflict of interest.

## Results

Of the 482 participants invited to take part in this study, 456 (94.6%) completed all questionnaire items. The mean age was 44.54 years (SD: 12.63). Most participants were male (75%), had up to 8 years of education (75%), were single (never married, 59.4%), had at least one child (53.7%), and migrated to São Paulo from other states in Brazil (52%). More than 30% of participants reported having a chronic health condition (lifetime), and 15.6% reported having been diagnosed with a sexually transmitted infection (lifetime). Regarding psychiatric characteristics, 49.6% reported depressive symptoms at clinical level (PHQ-9 ≥ 10 points), 55.7% reported using alcohol and other drugs (34.2%) at least once a week, in the last 30 days.

In terms of religious and spiritual characteristics, over 80% reported belief in a higher being and half had a nominal religion (50.4%). Over 30% attended a religious temple and the majority considered religion (52.4%) and spirituality (58.8%) to be very important in their lives. Table 1 shows the socio-demographic and clinical characteristics of the participants.

**Table 1:** Sociodemographic and clinical information of the participants (*n* = 456)

Variables	Mean	SD
Age	44.54	12.63
	**n**	**%**
Gender		
Male	342	75.0
Female	114	25.0
Education		
Never studied	114	25.0
Up to 8 years	342	75.0
Marital status		
Single	271	59.4
Married	39	8.6
Separared/Widower	146	32.1
Children		
Yes	245	53.7
No	211	46.3
Place of origin		
São Paulo (city)	122	26.8
São Paulo (countryside)	90	19.7
Other states in Brazil	237	52.0
Other countries	7	1.5
Diagnosed chronic illness (lifetime)		
Yes	172	37.7
No	285	62.3
Diagnosed sexually transmitted infection (lifetime)		
Yes	71	15.6
No	385	84.4
Alcohol use (at least once a week in the last 30 days)		
Yes	254	55.7
No	202	44.3
Drug use (at least once a week in the last 30 days)		
Yes	156	34.2
No	300	65.8
Depressive symptoms (PHQ-9 > 10 points)^a^		
Yes	226	49.6
No	230	50.4
Religion		
Yes	230	50.4
No	226	49.6
Belief in God or superior being		
Yes	373	81.8
No	83	18.2

^a^ PHQ-9: Patient Health Questionnaire-9 (PHQ-9)

We evaluated the overall mean scores of the sample for each of our variables, including the scores for depression, religiosity, religious and spiritual coping, as well as spiritual well-being. Table 2 shows the means, standard error and confidence interval of these scores, assessed respectively using the PHQ-9, P-DUREL, SRC and FACIT-Sp.

**Table 2:** Participants’ depression, religiosity, religious and spiritual coping, and spiritual well-being scores (*n* = 456)

Variable	Mean (SE)	CI 95%
Depression symptoms (PHQ-9)	9.91 (0.29)	9.27–10.56
Religiosity (P-Durel)		
Organizational (OR)	4.01 (0,08)	3.85–4.17
Non organizational (NOR)	3.26 (0.79)	3.10–3.41
Intrinsic (IR)	7.97 (0.17)	7.64–8.31
Religious Spiritual Coping (SRC)		
Positive SRC	2.55 (0.63)	2.44–2.67
Negative SRC	2.15 (0.04)	2.06–2.24
Spiritual Well-Being (FACIT-Sp)		
Significance	7.95 (0.18)	7.59–8.31
Peace	8.37 (0.16)	8.04–8.70
Faith	7.65 (4.44)	7.26–8.07
Total	23.98 (9.04)	23.10–24.83

Note: Functional Assessment of Chronic Disease Therapy for Spiritual Well-Being (FACIT); PHQ-9: Patient Health Questionnaire-9 (PHQ*-9*); Duke Religious Index (P-Durel); Spiritual and Religious Coping Scale (SRC). SE: Standard Error; CI: Confidence Interval; OR = organizational religiosity; SE = standard error; NOR = non-organizational religiosity; IR = intrinsic religiosity; SRC = spiritual religious coping

### Sociodemographic and Clinical Variables Predictors of Depressive Symptoms

To investigate sociodemographic and clinical variables associated with depressive symptoms in PEH, we conducted a series of linear regression analyses. Female gender, lower educational attainment, a lifetime diagnosis of a STI or chronic disease, and regular use of alcohol and other drugs were significant predictors of depressive symptoms. Conversely, PEH from São Paulo had a lower risk of experiencing depressive symptoms compared to other participants in the study. However, age was not a significant predictor in our sample. Table 3 presents these results by variable.”

**Table 3:** Unadjusted linear regression model: associations between sociodemographic and clinical variables and depressive symptoms^a^

Variable	B (SE)	*p*	*R*	*R* ^2^
Age (mean)	-0.045(0.02)	0.079	0.007	0.005
Gender (reference - male)	2.602(0.736)	< 0.001	0.027	0.025
Education (reference - has studied formally)	-1.194(0.33)	< 0.001	0.027	0.025
Place of origin (reference - other countries)	-0.919(0.36)	0.012	0.014	0.012
Reported Chronic Disease (reference - no)	2.916(0.652)	< 0.001	0.042	0.040
Reported STI^b^ (reference - no)	4.987(0.860)	< 0.001	0.069	0.067
Alcohol use^c^ (reference - no)	2.223(0.642)	0.001	0.026	0.024
Drug use^c^ (reference - no)	2.767(0.668)	< 0.001	0.036	0.034

^a^PHQ-9 score was used as the dependent variable for depressive symptoms; scores > 10 indicate clinically significant symptoms. ^b^STI: Sexually Transmitted Infection; ^c^At least three times per week

### Variables on Religiosity and Spirituality

Table 4 shows the unadjusted and adjusted linear regression models between the association of religiosity and spirituality with depressive symptoms in homeless people. The unadjusted models provide an initial view of the relationship between the variables, while the adjusted models consider the effect of other variables controlled in the model. In the unadjusted model, the results indicated that participants who attended a religious temple more than once a week, who reported that spirituality is very important in life, with higher levels of organizational religiosity and intrinsic religiosity were less prone to depression. In relation to the dimensions of spirituality, higher levels of meaning, peace and faith were associated with a lower propensity to depression. Negative SRC strategies were associated with a higher propensity to depression, while positive SRC strategies were associated with a lower propensity to depression. After the model was adjusted for sociodemographic and clinical variables (age, gender, schooling, origin, STIs, alcohol and drug use), most of the associations remained significant, except for the presence of religion or its attributed importance, as well as non-organizational religiosity.

**Table 4:** Evaluation of the predictive power of religiosity and spirituality in symptoms of depression reported by homeless people (*n* = 456)

	Crude Model				Adjusted Model			
Variable	B (SE)	*p*	*R*	*R*²	B (SE)	*p*	*R*	*R*²
Religion	-1.060(0.64)	0.010	0.006	0.004	-0.740(0.587)	0.208	0.199	0.185
Attends religious temple	-3.712(0.65)	< 0.001	0.065	0.063	-2.710(0.614)	< 0.001	0.230	0.216
Frequency of going to religious temple	-2.451(0.40)	< 0.001	0.074	0.072	-1.842(0.380)	< 0.001	0.236	0.223
Importance of religion	-1.174(0.40)	0.004	0.019	0.016	-0.621(0.374)	0.098	0.201	0.187
Importance of spirituality	-1.770(0,40)	< 0.001	0.040	0.038	-0.852(0.389)	0.029	0.205	0.191
P-Durel								
OR	-0.813(0.18)	< 0.001	0.043	0.041	-0.621(0.167)	< 0.001	0.220	0.206
NOR	-0.633(0,18)	0.001	0.025	0.023	− 0.0.313(0.177)	0.077	0.202	0.188
IR	-0.521(0.08)	< 0.001	0.080	0.078	-0.366(0.079)	< 0.001	0.233	0.219
SRC								
Positive SRC	-0.783(0.24)	0.002	0.022	0.020	-0.610(0.231)	0.009	0.209	0.194
Negative SRC	+ 1.733(0.306)	< 0.001	0.066	0.064	1.295(0.293)	< 0.001	0.230	0.216
FACIT-sp								
Significance	-0.759(0.07)	< 0.001	0.205	0.204	-0.580(0.071)	< 0.001	0.301	0.288
Peace	-0.831(0.07)	< 0.001	0.206	0.204	-0.640(0.077)	< 0.001	0.305	0.292
Faith	-0.163(0.07)	0.025	0.011	0.009	-0.132(0.066)	0.046	0.203	0.189

Note 1.: Functional Assessment of Chronic Disease Therapy for Spiritual Well-Being (FACIT); PHQ-9: Patient Health Questionnaire-9 (PHQ*-9*) score was used as the dependent variable for depressive symptoms; scores > 10 indicate clinically significant symptoms.; Duke Religious Index (P-Durel); Spiritual and Religious Coping Scale (SRC). SE: Standard Error; OR = organizational religiosity; SE = standard error; NOR = non-organizational religiosity; IR = intrinsic religiosity; SRC = spiritual religious coping

Note 2: Adjusted Model for the variables age, gender, education, state of origin, chronic diseases), sexually transmitted infections, alcohol use, and drug use (all variables with *p* < 0.10 in Table 3)

We present in Supplementary Table 1 the associations between sociodemographic and clinical variables, and positive and negative SRC scores. Significant group differences suggest specific factors influence strategy adoption. These findings improve understanding of the association between SRC and the study’s outcome—depressive symptoms.

## Discussion

Our study aimed to assess the associations between religiosity and spirituality (RS) and symptoms of depression in Brazilians PEH living in the city of São Paulo, also considering the impact of the participants’ sociodemographic and clinical conditions. We found a high frequency of depressive symptoms in approximately 50% of the participants. This rate is significantly higher than that observed in the general population of São Paulo, which was 16.9% in a large epidemiological study [11]. This finding is consistent with the literature, which indicates a four to five times greater likelihood of depression among homeless people compared to the general population [13]. Other studies corroborate these results, showing high rates of depression PEH in Belo Horizonte [35] and Rio de Janeiro [36], as well as in international systematic reviews [2, 37]. We used the PHQ-9 to assess depressive symptoms, a self-report instrument with high sensitivity and specificity for diagnosing Major Depression [34]. The consistency of these findings with the literature reflects the validity of our method.

### Impact of Sociodemographic and Clinical Conditions

The results show that sociodemographic and clinical characteristics of the participants predict depressive symptoms. Previous studies have identified a high frequency of depressive symptoms among PEH aggravated by factors such as gender, substance abuse, the presence of chronic diseases and sexually transmitted infections, as well as other mental health problems [39, 40]. The interaction between these factors exacerbates psychological distress, as chronic health conditions and substance abuse often lead to worsening mental health, while stigma and lack of healthcare access further perpetuate depressive symptoms. The lack of statistical significance of the association between age and depressive symptoms may be attributed to a homogeneous age distribution in the sample, or to the more direct and immediate influence of street living conditions and limited access to health resources on depressive symptoms. Unique adversities, such as housing instability, violence, social isolation and lack of access to basic services, have a more marked influence than age itself [17, 31, 41]. These results highlight the complexity of PEH experiences and the need for integrated approaches that consider socioeconomic and clinical factors in understanding depressive symptoms.

### Impact of Religiosity and Spirituality

We found significant associations between RS and depressive symptoms, even after adjusting for sociodemographic and clinical factors. Participation in religious activities, positive religious coping and the importance attributed to spirituality were associated with a lower propensity to depression. Specific dimensions of religiosity, such as organizational religiosity (OR) and intrinsic religiosity (IR), and spirituality, such as meaning, peace and faith, also showed significant associations with lower depression. These findings are in line with previous studies highlighting the protective role of CR in mental health, including reduced suicidal ideation and substance use [42–44].

Frequent participation in religious institutions has emerged as a prominent factor in reducing depression [26]. Regularly attending a religious institution can contribute to the reduction of depressive symptoms through belief in a higher being, a reduction in the sense of uncontrollability, the construction of a routine, an increase in the social support network and a sense of belonging. Attending a religious institution that welcomes and cares for physical, emotional and spiritual needs can mitigate the harmful effects of living on the streets, while also reducing its impact on mental health [45, 46].

Dysfunctional forms of SRC have been associated with a greater propensity for depressive symptoms. Behaviors such as religious interpersonal conflicts, blaming, punishment and dissatisfaction with religious entities exacerbate depressive symptoms [21, 26, 47]. Religious conflicts manifest themselves in various dimensions, including interpersonal conflicts with religious communities, divine disagreements and intrapersonal dilemmas [48]. A meta-analysis highlighted a significant correlation between religious conflict and negative mental health outcomes such as depression, anxiety and suicidal tendencies [49]. The pronounced effect of negative SRC can be interpreted as a manifestation of the deleterious effects of religious conflict on mental health [50].

Having a specific religion and attributing importance to it were not significant after adjusting for sociodemographic and clinical variables, highlighting the complexity of this association. These findings agree with studies that highlight the multifaceted and sometimes ambivalent role of religion and spirituality in mental health [51, 52]. For some people, religion offers comfort and hope; for others, it can represent sources of guilt and conflict [53, 54].

### Implications

Our study has significant implications for the scientific community, expanding knowledge about the role of RS in the mental health of homeless people, a population historically underrepresented in research, especially in developing countries. By investigating the relationship between RS and depression in homeless people, our research deepens the understanding of the underlying mechanisms that influence mental health in this adverse context. Furthermore, it reinforces that public health policies and social intervention programs should recognize the role of RS in promoting social reintegration, alongside physical and emotional needs. This can include partnerships with local religious organizations to provide emotional support, practical assistance and access to basic resources.

### Limitations and Future Directions

Although we have achieved our objective, some limitations need to be presented. The cross-sectional design does not allow us to establish causal relationships between RS and depression. We recommend longitudinal studies for this purpose. Data collection was limited to one region of São Paulo. Although we evaluated a comprehensive sample of PEH in São Paulo, the results may not represent other groups in the city, given the cultural diversity. It is necessary to replicate the study in different regions of São Paulo and in other Brazilian capitals to assess possible specificities.

Although we used validated instruments, such as the PHQ-9, to assess depressive symptoms, and RS measures, instruments in which participants report their perceptions of psychosocial and psychopathological phenomena, such as those investigated in the present study, can be influenced by social desirability bias and each individual’s capacity for self-perception. Therefore, it is possible that less evident symptoms of depression may have been overlooked by participants. We believe, therefore, that clinical assessment by a mental health professional could identify symptoms of depression at an even higher frequency than that identified in our study. We suggest mixed-method studies combining quantitative and qualitative data for a more comprehensive understanding of associations between RS and depressive symptoms among PEH. Qualitatively assessing the practical effects of RS on behavior change and life narratives may provide additional insights.

## Conclusion

Our results suggest that RS can both protect and harm the mental health of homeless individuals. Positive SRC strategies should be integrated into interventions for this population, as they offer significant mental health benefits. In contrast, negative SRC, often referred to as “red flags,” should be closely monitored by health professionals due to its potentially harmful effects, such as intensifying feelings of guilt, conflict, or distress [55]. We believe that professionals should remain alert to these warning signs and collaborate with trained religious leaders to adopt a more holistic and effective approach to treating depression. While incorporating religious and spiritual aspects is essential, this should be paired with practical measures that ensure dignity, such as access to housing, adequate nutrition, and employment. Ultimately, RS should be recognized as a vital component of clinical practice [56], especially when addressing the complex needs of vulnerable populations like PEH.

## Electronic Supplementary Material

Below is the link to the electronic supplementary material.

Supplementary Material 1

## Data Availability

Data will be available upon request to the first author (email: felipe.carvalho@ubi.pt).
