# Supplementary material for: Associations Between Religiosity, Spirituality and Depressive Symptoms Among People Experiencing Homelessness in São Paulo, Brazil: An Observational Study
Source: Psychiatr Q. 2025 Jun 9;97(1):185–201. doi: 10.1007/s11126-025-10163-5 (PMC13033023; doi:10.1007/s11126-025-10163-5)
Supplement: Supplementary file 1 — Supplementary Material 1 [file 11126_2025_10163_MOESM1_ESM.docx]

**Supplementary Table1** *Associations Between Sociodemographic and Clinical Variables and Positive and Negative Spiritual Religious Coping*

| **Variable** | **Positive SRC** | ***p*** | **Negative SRC** | ***p*** |
| --- | --- | --- | --- | --- |
| Age^a^ (mean) | r= -0.100 | 0.033 | r=0.371 | <0.001 |
| Gender^b^  Female  Male | 2.81 (1.26)  2.47 (1.30) | 0.013 | 2.44 (0.88)  2.05 (1.04) | <0.001 |
| Education^b^  Never studied  Up to 8 years | 2.54 (1.26)  2.56 (1.31) | 0.883 | 2.22 (0.97)  2.13 (1.03) | 0.391 |
| State of origin^c^  São Paulo (capital)  São Paulo (interior)  Other states in Brazil  Other countries | 2.67 (1.32)  2.66 (1.22)  2.44 (1.30)  3.08 (3.08 | 0.211 | 2.28 (1.00)  2.34 (0.90)  1.98 (0.95)  3.10 (0.39) | <0.001 |
| Reported Chronic Disease^b^  Yes  No | 2.87 (0.95)  2.36 (1.31) | <0.001 | 2.48 (0.99)  2.01 (1.01) | <0.001 |
| Reported STI^b^  Yes  No | 2.82 (1.16)  2.50 (1.31) | 0.040 | 2.51 (0.90)  2.09 (1.00) | 0.001 |
| Alcohol use^b,d^  Yes  No | 2.39 (1.27)  2.76 (1.30) | 0.002 | 2.62 (1.09)  2.02 (1.02) | <0.001 |
| Drug use^b,d^  Yes  No | 2.04(1.05)  2.85 (1.11) | <0.001 | 2.77 (0.99)  2.19 (1.08) | 0.001 |

^a^Pearson Matrix Correlation; ^b^ t test; ^c^One-Way ANOVA; ^d^At least three times per week.

SRC: SRC: Spiritual Religious Coping; STI: Sexually Transmitted Infection.
